# TRIAMF: A New Method for Delivery of Cas9 Ribonucleoprotein Complex to Human Hematopoietic Stem Cells

**DOI:** 10.1038/s41598-018-34601-6

**Published:** 2018-11-02

**Authors:** Jonathan Yen, Michael Fiorino, Yi Liu, Steve Paula, Scott Clarkson, Lisa Quinn, William R. Tschantz, Heath Klock, Ning Guo, Carsten Russ, Vionnie W. C. Yu, Craig Mickanin, Susan C. Stevenson, Cameron Lee, Yi Yang

**Affiliations:** 1 Chemical Biology and Therapeutics, Novartis Institutes for BioMedical Research, Cambridge, Massachusetts USA; 2 NIBR Informatics, Novartis Institutes for BioMedical Research, Cambridge, Massachusetts USA; 3 Biotherapeutic and Analytical Tech, Novartis Institutes for BioMedical Research, Cambridge, Massachusetts USA; 4 Biotherapeutics & Biotechnology, The Genomics Institute of the Novartis Research Foundation, La Jolla, California USA; 5 Global Discovery Chemistry, Novartis Institutes for BioMedical Research, Cambridge, Massachusetts USA

## Abstract

CRISPR/Cas9 mediated gene editing of patient-derived hematopoietic stem and progenitor cells (HSPCs) *ex vivo* followed by autologous transplantation of the edited HSPCs back to the patient can provide a potential cure for monogenic blood disorders such as β-hemoglobinopathies. One challenge for this strategy is efficient delivery of the ribonucleoprotein (RNP) complex, consisting of purified Cas9 protein and guide RNA, into HSPCs. Because β-hemoglobinopathies are most prevalent in developing countries, it is desirable to have a reliable, efficient, easy-to-use and cost effective delivery method. With this goal in mind, we developed TRansmembrane Internalization Assisted by Membrane Filtration (TRIAMF), a new method to quickly and effectively deliver RNPs into HSPCs by passing a RNP and cell mixture through a filter membrane. We achieved robust gene editing in HSPCs using TRIAMF and demonstrated that the multilineage colony forming capacities and the competence for engraftment in immunocompromised mice of HSPCs were preserved post TRIAMF treatment. TRIAMF is a custom designed system using inexpensive components and has the capacity to process HSPCs at clinical scale.

## Introduction

β-hemoglobinopathies are the most common monogenic blood disorders caused by a faulty β-hemoglobin gene, which encodes one of the two subunits of adult hemoglobin (HbA, α_2_β_2_). The two major forms of β-hemoglobinopathies are β-thalassemia and sickle cell disease (SCD). SCD is more severe and affects over 300,000 newborns a year globally and more than 70% of these new cases are in Sub-Saharan Africa^1,2^. Unlike β-thalassemia, which is caused by insufficient production of β-hemoglobin, SCD is caused by a single adenine to thymine transversion at the seventh codon of the β-globin gene, which converts a hydrophilic glutamate to a hydrophobic valine. The mutant hemoglobin (HbS) polymerizes under hypoxic conditions leading to “sickling” of the red blood cells (RBC). The sickled RBC become rigid with a significantly reduced life span and tend to clog capillaries, which lead to clinical manifestations of SCD including stroke, nephropathy, acute chest syndrome, infections, pain crises and anemia. There are limited treatment options for β-hemoglobinopathies to date. Allogeneic hematopoietic stem cell transplantation (HSCT) can be curative but this option is limited by the availability of matched donors and the risk of graft-vs-host disease^3^.

The clearly defined genetic defect has made β-hemoglobinopathies the ideal targets for gene therapy. One approach for treating both β-thalassemia and SCD is to reactivate the post-natal silenced γ-globin (HBG) gene expression in adult RBCs. This is based on a long-known observation that β-hemoglobinopathy patients carrying concomitant mutations that result in sustained fetal globin (α2γ2, HbF) expression (hereditary persistence of fetal hemoglobin, or HPFH) have attenuated symptoms^4^. In addition, the benefit from hydroxyurea treatment for certain patients has been mainly attributed to its potency for inducing HbF expression^5,6^. In this context, several strategies have been investigated to achieve induction of HbF by genetic manipulation of patient-derived HSPCs for autologous transplantation^7–14^. Recently CRISPR/Cas9 mediated gene editing was successfully applied to recapitulate a naturally occurring HPFH mutation in CD34^+^ HSPCs leading to elevated HbF expression in RBCs derived from edited cells *in vitro*^15^. However lentiviral or DNA plasmid based vectors were used in that study for delivery of CRISPR/Cas9 into HSPCs, which could increase the risk of off-target editing due to prolonged exposure of cells to CRISPR/Cas9^16,17^.

RNP complexes consisting of purified Cas9 protein and synthetic single gRNA (sgRNA) have been established as the preferred format for delivery of CRISPR-Cas9 *in vitro* and *in vivo* due to its fast editing kinetics, increased efficiency, enhanced selectivity and improved cell viability^16–19^. Although numerous methods have been explored for efficient delivery of RNPs into different cell types including iTOP^20^, nanoparticles^21–24^, cell penetrating peptides^25,26^ and lipids^27,28^, none of these methods has been successfully applied for delivery of RNPs into HSPCs. This might be at least partly due to the fact that these methods rely on endocytosis pathways, which for HSPCs are very different from the cell lines used for developing these methods^29^. To date electroporation remains the primary choice for RNP delivery into HSPCs^30,31^, but electroporation of RNPs into HSPCs at a clinical scale has not been reported.

Cell membrane deformation via microfluidics devices has been shown to be an effective method for intracellular delivery of a variety of biomolecules including RNPs^32–34^. These devices rely on a microfabricated chip that is primarily designed for research purposes and more suitable for processing small amount of cells due to a tendency to clog^34,35^. In order to apply the concept of using cell constriction for intracellular delivery of biomolecules but to overcome the scale limitations of the reported methods, we developed TRIAMF, a filter membrane based cell permeabilization device as a new low cost and non-electroporation based delivery system that can effectively and safely deliver RNPs to CD34^+^ HSPCs at large scale.

## Results

### Optimization of conditions for delivery of RNP into primary human CD34^+^ HSPCs using TRIAMF

**Figure 1:**
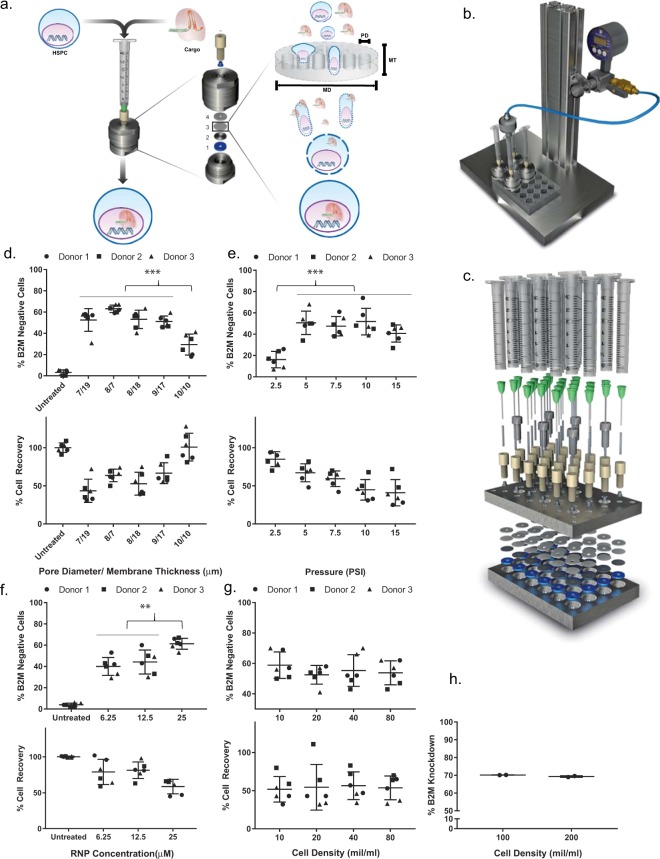
Design and optimization of TRIAMF. (**a**) Schematic illustration of the single unit system. The device consists of a silicone washer (1), a stainless steel mesh (2), a hydrophilic track-etched polycarbonate filter membrane (3), and a PTFE washer (4). MD: membrane diameter; MT: membrane thickness; PD: pore diameter. Not drawn to scale. (**b**) Illustration of basic set up, including the pressure regulator and valve from a nitrogen source, connector, and the membrane holder. (**c**) Illustration of a 24-membranemanifold system. B2M knockout efficiency determined by FACS and cell recovery as a function of (**d**) membrane pore diameter and thickness; (**e**) applied pressure; (**f**) RNP concentration; (**g**) cell density (n = 6, 3 donors with duplicate of each donor). When not specified, the sample volume was kept at 50 μl, cell density at 8 × 10^7^ cells/ml, nitrogen pressure at 5 PSI and 7 μm thick membranes with 8 μm pore diameter were used. (**h**) B2M knockout efficiency remained the same with further increased cell density from 10^8^ to 2 × 10^8^ cells/ml in 50 μl volume processed by TRIAMF using 25 μM of RNP, 7 μm thick membrane with 8 μm pore diameter under 5 PSI nitrogen pressure (n = 2). ***p < 0.001, **p < 0.002, one-way analysis of variance (ANOVA) and Tukey’s multiple comparison test. Bars represent standard deviation. Alan Abrams helped to produce the graphics in (**a**–**c**).

It was reported by Miller’s lab in the late 90s that fluorescent dextran of molecular weight up to 500,000 Da could be delivered inside CHO cells by passing the dextran/cell suspension through a filter membrane^36^, but it was unknown whether the technique could be applied to HSPCs and/or to the delivery of macromolecular complexes such as RNPs. We decided to test the feasibility of transducing HSPCs with RNPs by passing a mixture of RNP and HSPCs through a filter membrane. The design principle for TRIAMF is illustrated in Fig. 1a. Once inside the cell, the nuclear localization signals from the Cas9 protein should be able to direct RNP into the nucleus. To reduce the cost of purchasing both RNP and CD34+ HSPCs, we fabricated a customized filter holder with a minimized internal dead volume. It allows efficient recovery of an input of 50 μl liquid, one tenth of the volume required for most commercially available filter holders (Fig. 1a). The device consists of a silicone washer, a stainless steel mesh, a hydrophilic track-etched polycarbonate filter membrane, and a PTFE washer. The filter holder is connected to a 3 ml syringe, which serves as a reservoir for the RNP/HSPC mixture. After loading the sample into the tip of the syringe, it is sealed with a connector from a nitrogen regulator (Fig. 1b). The RNP/HSPC mixture is then pushed through the membrane holder by nitrogen pressure in an instant and collected directly into tissue culture plates. This device can be cleaned by sonicating the parts in 70% isopropanol and can be reassembled with a new membrane. To facilitate higher-throughput experimentation, we also built a 24-membrane manifold that allows testing up to 24 delivery conditions in a single run with just one assembly step (the flow-through are collected directly into a 24-well tissue culture plate) (Fig. 1c).

To establish the optimal conditions for TRIAMF, we used the 24-membrane manifold to systematically test the parameters that might influence both the RNP delivery efficiency and cell recovery including membrane thickness and pore diameter, nitrogen pressure applied to the system, RNP concentration and cell density. We measured surface expression of β2-microglobulin (B2M) as the readout to evaluate the delivery efficiency of RNP containing a B2M targeting gRNA into HSPCs^37^. In each of the following experiments, we optimized the aforementioned variables one at a time while the rest remained constant. We first tested all available filter membranes with different membrane thickness and pore diameter to process 50 μl of HSPCs at 8 × 10^7^ cells/ml with 25 μM of RNP (Cas9:sgRNA molar ratio = 1:1, Materials & Methods) using 5 pounds per square inch (PSI) nitrogen pressure. We found that a 7 μm thick membrane filter with 8 μm pore diameter gave the highest B2M knockout (63.1% ± 3.5% by FACS, n = 6, Fig. 1d) with a cell recovery of 63.7% ± 5.6% (Fig. 1d). Increasing the pore diameter to 10 μm reduced B2M knockout by almost half, though the cell recovery was improved (Fig. 1d). The average diameter of CD34^+^ HSPCs before TRIAMF treatment was 12 μm and there was no obvious change in cell size distribution post TRIAMF treatment, suggesting that TRIAMF does not selectively eliminate large cells (Fig. S1). Furthermore the size distributions of either CD34^+^ or CD34^+^ CD90^+^ cells were similar between untreated cells and TRIAMF treated cells in the absence (TRIAMF Mock) or presence of RNP (TRIAMF + RNP, Fig. S1). Finally we found that the B2M knockout level in CD34^+^ HSPCs or the more primitive CD34^+^/CD90^+^ HSCs^38,39^ was similar as in the total cell population, suggesting that RNP was delivered indiscriminately into these subpopulations with similar efficiency (Fig. S2).

Next we optimized the nitrogen air pressure used to push the cells through a 7 μm thick membrane filter with 8 μm pore diameter. As shown in Fig. 1e, using a nitrogen pressure of 5 PSI struck the optimal balance between B2M knockout and cell recovery for processing 50 μl of HSPCs at 8 × 10^7^ cells/ml mixed with 25 μM of RNP. We then tested whether we could reduce the concentration of RNP without compromising editing of the same amount of HSPCs and found that 25 μM of RNP led to maximum B2M knock out at 61.3% ± 4.6% with cell recovery at 58.7 ± 11% (Fig. 1f).

Lastly, we checked whether RNP could be delivered in mixtures comprising high cell concentrations. Interestingly, we found that both B2M knockout and cell recovery remained constant within the range of cell densities tested (Fig. 1g), suggesting that the system has the capacity for high-concentration, large-scale cell processing. Similar conclusions about the optimal parameters were confirmed with on-target Next Generation Sequencing (NGS) (Fig. S3).

We explored the scalability of the TRIAMF system using the single manifold system to pass 50 μl of RNP/HSPC mixture through a 7 μm thick membrane with 8 μm pore diameter under 5 PSI nitrogen pressure. With the RNP concentration fixed at 25 μM, we were able to obtain a similar B2M knockout efficiency with increased cell densities of 10^8^/ml to 2 × 10^8^/ml (Figs 1h and S3). The cells were able to pass through the system instantly with no indications of clogging.

To determine the impact of TRIAMF treatment on CD34^+^ HSPC expansion *ex vivo*, we compared the growth rate of cells treated with either TRIAMF or electroporation in the absence of RNP to exclude the potential effects from gene editing. We either electroporated HSPCs (Neon Mock) or treated HSPCs with TRIAMF (TRIAMF Mock) and let the cells recover for 48 hours. As the control group for electroporation, cells were mixed with the electroporation buffer but not electroporated (Neon Untreated). As the control group for TRIAMF, cells were treated identically to the TRIAMF Mock group except without passing through the membrane (TRIAMF Untreated). On the third day post-treatment, we seeded the same number of viable cells from each experimental group and expanded these cells for 7 days. We found that electroporation resulted in a reduced total cell number at day 7 post-treatment with proportionally reduced CD34^+^ or CD34^+^/CD90^+^ cell numbers (Fig. S4). On the other hand, TRIAMF treated cells showed similar expansion capacity with no obvious change in CD34^+^ or CD34^+^/CD90^+^ population compared to the untreated cells (Fig. S4).

### TRIAMF treatment does not impair multilineage potential and engraftment in NSG mice

**Figure 2:**
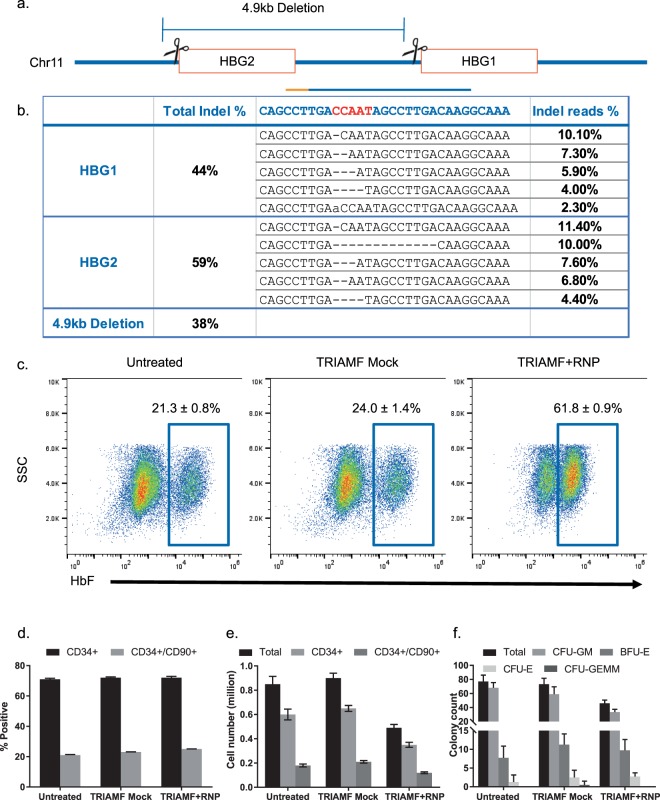
Genome editing of the HBG1 and HBG2 promoters using TRIAMF mediated delivery of RNPs. (**a**) HBG1 and HBG2 promoter and g1-RNP cutting sites. (**b**) The sequence of HBG1 and HBG2 target sites, the bar indicates targeting gRNA-1 with orange indicating the PAM site. The putative transcription repressor binding CCAAT box sequence is marked in red. Total indel percentages among total reads by NGS with top 5 frequent alleles. The 4.9 kb deletion between the two target sites as detected by qPCR. (n = 2, single donor, biological duplicate, indel patterns are mean of biological duplicates). (**c**) Representative flow cytometry plots showing HbF^+^ immunostaining of CD235a^+^/CD71^−/low^ cells after erythroid differentiation for 3 weeks of untreated, mock treated (TRIAMF Mock), and g1-RNP edited (TRIAMF + RNP) HSPCs (see Fig. S5 for gating strategy). (**d**) Immunostaining of HSPCs for CD34 and CD90 markers after 7 day expansion post treatment. (**e**) Cell count of CD34^+^ and CD34^+^/CD90^+^ cells after 7-day expansion post treatment. (**f**) Colony forming unit assay. Numbers indicate mean +/− SD, n = 4 (single donor, biological duplicate with technical duplicate each).

We then tested the feasibility of applying TRIAMF for delivery of RNPs into HSPCs to induce HbF expression. We chose a guide RNA previously reported by Traxler *et al*. that recapitulates a naturally occurring 13 base pair deletion found in a group of SCD patients with HPFH^15^. Using the optimized TRIAMF protocol, we transduced *ex vivo* expanded bone marrow-derived CD34^+^ HSPCs from healthy donors with RNPs consisting of the gRNA-1 described by Traxler *et al*.^15^. This gRNA has two identical target sites in the HBG loci^15^ (Fig. 2a). As experimental controls, cells were either left untreated or TRIAMF-treated in the absence of RNPs (TRIAMF Mock). We then split the cells from each experimental group into two aliquots and collected one aliquot 48 hours post treatment for on-target indel analysis using NGS. We detected an average of 44% indels at the HBG2 site and 33% at the HBG1 site (Fig. 2b). In addition, we were also able to quantify up to 38% of mutant alleles containing the 4.9 kb deletion between the two on-target sites using qPCR as described by Traxler *et al*.^15^.

The other aliquot of cells was differentiated into erythrocytes and quantified for HbF expression within mature erythrocytes defined by erythroid maturation markers CD235a^+^/CD71^−/low^ using flow cytometry (Figs 2c and S5). We detected 21%, 23%, and 60% of HbF+ cells in untreated, mock treated (TRIAMF Mock), and RNP treated cells (TRIAMF + RNP) respectively with a net ~40% induction of HbF+ cells over background, similar to what Traxler *et al*. reported^15^ (Fig. 2c).

To assess the impact of TRIAMF treatment on CD34^+^ HSPC expansion *ex vivo*, we cultured 5 × 10^4^ cells that had undergone different treatments for 7 days and compared the number and percentage of CD34^+^ and CD34^+^/CD90^+^ cells from each group at the end of the experiment. We found that the total cell number and the percentage of CD34^+^ or CD34^+^/CD90^+^ remained the same between untreated and mock treated (TRIAMF Mock) groups (Fig. 2d,e). However there was an obvious reduction of both populations in the g1-RNP treated group (TRIAMF + RNP, Fig. 2e).

Next we evaluated the impact of TRIAMF on the multilineage differentiation potential of HSPCs using the colony forming assay. We found that the number and composition of lineage defined colonies were indistinguishable between untreated and mock treated cells (TRIAMF Mock) but the total number of colonies derived from g1-RNP treated cells (TRIAMF + RNP) was noticeably reduced (Fig. 2f).

**Figure 3:**
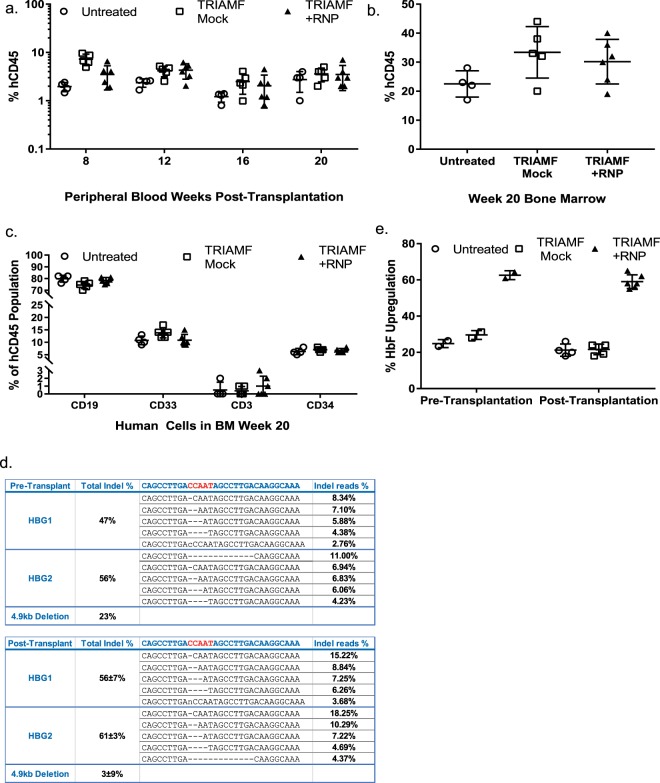
Preservation of engraftment competency of HSPCs post TRIAMF treatment. 7 × 10^5^ viable HSPCs (from the same donor as used in Fig. 2) 1-day post treatment were transplanted into each sub-lethally irradiated recipient mouse via tail vein injection. (**a**) Human CD45^+^ chimerism of untreated (n = 4 mice), mock treated (TRIAMF Mock, n = 5 mice), and g1-RNP edited (TRIAMF + RNP, n = 6 mice) in the peripheral blood 8, 12, 16, and 20 weeks post-transplantation. (**b**) Human CD45^+^ chimerism in mouse bone marrow 20 weeks post-transplantation. (**c**) Lineage distribution of human CD45^+^ cells isolated from mouse bone marrow 20 weeks post-transplantation. (**d**) Two aliquots of HSPCs from the same donor were processed by TRIAMF. One aliquot of cells was harvested 48 hours after treatment (Pre-Transplant, n = 1) and the other aliquot of cells were engrafted into NGS mice from which hCD45^+^ cells were harvested from mouse bone marrow 20 weeks after transplantation (Post-Transplant, n = 6 mice). The total mutation rate and top 5 most frequent indel alleles as a percentage of total NGS reads at HBG1 and HBG2 sites were shown. For the Post-Transplant group, indel patterns are the mean of all the mice. (**e**) Comparison of HbF^+^ immunostaining of CD235a^+^/CD71^−/low^ cells after erythroid differentiation of HSPCs 48 hours post treatment (n = 2, technical duplicate) and hCD45^+^/CD34^+^ HSPCs harvested from mouse bone marrow 20 weeks post transplantation (n = 4–6 mice).

To determine the long-term engraftment potential of TRIAMF treated HSPCs, we injected untreated HSPCs and TRIAMF treated HSPCs with or without RNPs into sub-lethally irradiated non-obese (NOD)/severe combined immunodeficiency (SCID)/*Il2rg*^−/−^ (NSG) mice. We observed comparable human chimerism reconstituted from different treatment groups as determined by the presence of hCD45^+^ cells either in circulation at 8, 12, 16 and 20 weeks or the bone marrow 20 weeks post-transplantation (Fig. 3a,b). These engraftment frequencies are comparable to those measured at 16 weeks after transplantation of HSPCs treated with different gene editing methodologies^9,40^. Furthermore there was no significant difference in the multilineage reconstitution capability between untreated and mock treated HSPCs (ANOVA one-way analysis of variance). Surprisingly g1-RNP edited HSPCs engrafted equally well (Fig. 3c) despite impaired expansion and differentiation potential *in vitro* (HSPCs from the same donor were used in this experiment as shown in Fig. 2b–f).

At 20 weeks following transplantation, we sorted hCD45^+^ cells harvested from mouse bone marrow and determined the on-target indel frequency and pattern at HBG1 and HBG2 sites in these cells. We found that they were comparable with those of the input cells pre-transplantation (Fig. 3d). Interestingly we found the frequency of alleles containing the 4.9 kb deletion was decreased in the hCD45^+^ cells recovered from mouse bone marrow 20 weeks after transplantation (Fig. 3d). In addition, the frequency of the 13 bp deletion was reduced from 11% to 4%. The reproducibility and significance of these changes remain to be determined. Despite these changes CD235a^+^/CD71^−/low^ erythrocytes derived from hCD34^+^ cells isolated from the bone marrow of each mouse showed an average of 40% induction of HbF^+^ cells above background (Fig. 3e), consistent with the number of HbF^+^ CD235a^+^/CD71^−/low^ erythrocytes differentiated directly 48 hours after treatment (Fig. 2c).

## Discussion

The promise of using RNP edited HSPCs for treating monogenic disorders has motivated us to develop an efficient, low-cost, non-viral and non-electroporation based method for RNP delivery. In addition to electroporation, other biophysical methods such as sonication and hypotonic permeabilization have been used to deliver biomolecules to cells^41^. We were particularly attracted to a previously reported observation that when a cell passes through a space smaller than its size, its cellular membrane gets stretched, resulting in the formation of transient “pores” that allow extracellular biomolecules to passively diffuse inside the cell^32,34,36,42^. We reasoned that this strategy could be particularly suited for HSPCs because of their innate ability to navigate through spaces smaller than their size within the bone marrow niche via transendothelial migration^43–46^. We developed TRIAMF based on this concept and systematically optimized conditions to deliver RNPs into HSPCs for robust gene editing at two therapeutically relevant loci. Our data supports the hypothesis that HSPCs are susceptible to membrane deformation based permeabilization. Whether the mechanism for TRIAMF mediated biomolecule delivery into HSPCs is similar to what has been reported by Sharei *et al*.^47^ remains to be determined.

Compared with TRIAMF, electroporation of the same RNP complex targeting B2M into HSPCs at a much smaller scale using the Neon electroporator led to higher editing efficiency but the cell recovery rate was significantly compromised (Fig. S6). This difference seems not due to increased B2M knockout efficiency because cells treated with mock electroporation also had a slower recovery rate than the cells treated with mock TRIAMF (Fig. S4). Interestingly there has been no report detailing the potential impacts of the electroporation buffers to HSPCs. In this regard, TRIAMF can be performed directly in HSPC culture medium and does not require specialized electroporation buffer. As of the writing of this manuscript, there is only one commercially available electroporator capable of clinical scale cell processing. It remains to be determined how this instrument performs against TRIAMF in the future.

Compared with the microchip based cell constriction methods reported in the literature, we think TRIAMF is simpler, cheaper and easier to scale up without the need for sophisticated microfluidics fabrication. The filter membranes used in TRIAMF contains 10^5^ parallel pores per cm^2^. The membrane surface area through which the cells pass in the device we used in this report is 0.0706 cm^2^ (calculated based on the inner diameter of the O-ring) and contains ~7000 total parallel pores. This number far exceeds the 45 microfluidic channels per chip described in a recent report, which usually clogs after processing 1 million cells^34^. Due to the cost of HSPCs, the maximum density we tested was 2 × 10^8^/ml cells in a 50 μl sample volume. We envision that we can either increase the volume of cells processed or increase the membrane surface area through which the cells pass (or both) to scale up the amount of cells processed. Additionally, with the simplicity of TRIAMF, it can be adapted into a single use disposable cartridge for clinical or research use.

Using TRIAMF, we achieved similar editing efficiency and HbF-induction reported by Traxler *et al*. *in vitro* and demonstrated that TRIAMF does not impair the multilineage and engraftment potential in NSG mice. Of note, Traxler *et al*. reported an equal proportion of modification at both sites and the 4.9 kb deletion was only detected in a few edited HUDEP-2 clones but not in edited primary human HSPCs^15^. Although we are not certain the reason for these disparities, our data demonstrated that the erythrocytes derived from the engrafted HSPCs maintained high level HbF induction 20 weeks post-engraftment.

In summary, TRIAMF is a simple and robust method for delivery of RNPs into HSPCs particularly suited for clinical scale processing. The utility of this method for delivery other biomolecules in hard-to-transfect cell types remains to be determined.

## Methods

### Material

#### TRIAMF System Set Up

Stainless steel devices (single and 24 well manifold) were custom manufactured by Industrial Motions Engineering. Stainless steel disc membrane supports were manufactured by Laser Services. All other parts were purchased from McMaster Car. Track etched polycarbonate PVP coated membranes were purchased from Sterlitech with varying pore sizes (defined by pore diameter) and thicknesses (item number PCT8013100 for 7 μm thick membrane with 8 μm pore diameter; PCT10013100 for 10 μm thick membrane with 10 μm pore diameter. Other membranes used in this report were custom-made).

Silicone O-ring was placed, followed by stainless steel mesh, followed by polycarbonate membrane (shiny side up), followed by Teflon washer, the top was attached and tightened (24-manifold via screws, single unit via wrenches). 3 ml syringe was then attached to the needle assembly.

Prior to transfection, the manifold was calibrated by running 2 ml of sterilized DI water, followed by 1 ml of 70% ethanol and then followed by another 2 ml of sterile DI water at 5 psi.

#### Human hematopoietic stem and progenitor cells culture

All human tissues used in this study received informed donor consent and their usage was tracked internally by Novartis Institutes for BioMedical Research (NIBR) Human Tissue Network. All experimental protocols involving human tissues were approved by NIBR Human Tissue Council, and all experiments involving human tissues were executed in full compliance to NIBR Human Tissue Council Research and Quality guidelines. Cryopreserved bone marrow derived CD34^+^ HSPCs were purchased from Lonza or AllCells and thawed according to vendors’ instructions. Cells were cultured at 37 °C 5% CO_2_ in SFEM II supplemented with CC110 (StemCell Technologies), 0.75 μM StemRegenin 1 (StemCell Technologies), 50 nM UM171 (StemCell Technologies), 50 ng/mL human recombinant IL-6 (Peprotech), and Pen-Strep (Life Tech 15140-122). Cells were cultured for 3–5 days before being used in experiments.

#### RNP Delivery via TRIAMF

Wild type recombinant *S*. *pyogenes* Cas9 protein with two SV40 nuclear localization signals and one 6xHis-tag is expressed in *E*.*coli* and purified based on published protocols^48^. The sgRNAs were purchased from AxoLabs. The B2M targeting domain sequence is GGCCGAGAUGUCUCGCUCCG (crB2M_6 by Mandal *et al*.^37^) and the gRNA-1 is CUUGUCAAGGCUAUUGGUCA.

*Ex vivo* expanded human HSPCs were spun down and resuspended in 20 μl of SFEM II media. 200 μg of Cas9 (6.3 mg/ml) was mixed with 40 μg of sgRNA (10 mg/ml) (1:1 molar ratio) and allowed to complex by incubating for 5 minutes at room temperature. The RNP mixture was mixed with the cells and allowed to incubate for 2 minutes at RT with a final volume of 50 μl, in which the concentration of RNP and cell density were calculated and described in this report. The mixture was then transferred into the tip of the syringe connector. Then the mixture was forced through the syringe and membrane via 5 PSI of nitrogen pressure.

The flow-through was allowed to rest for approximately 2–5 minutes while the membrane was washed with 1 ml of complete media. The cells were then supplemented with fresh media and cultured for 48–72 hours before analysis.

#### Cell Lysis Prep for Next Generation Sequencing

Editing efficiency was determined via NGS as previously described^49^. Approximately 10^5^ cells were spun down and lysed with 40 μl a lysis buffer (10 mM Tris-HCl, pH 8.0; 0.05% SDS) with proteinase K at final concentration of 100 μg/ml (EO0491, Thermo Scientific). 2 μl of the cell lysis extract was directly used to amplify target sequence via primers (B2M forward: 5′-ctctcaaaccacagggatcaca; B2M reverse: 5′-ctccatcaccaagagagcctt; HBG1 forward: 5′-cgctgaaactgtggctttatagaaatt; HBG2 forward: 5′-gcactgaaactgttgctttataggat; HBG1/2 reverse: 5′-ggcgtctggactaggagcttattg) and Platinum Taq polymerase (Clontech).

#### Comparison of the expansion of Mock treated HSPCs by Neon Electroporation and TRIAMF

2 × 10^5^ *ex vivo* expanded human HSPCs were collected by centrifugation, washed once with PBS, and resuspended in 10 μl of Neon electroporation buffer T (Invitrogen, MPK1096Tb). The HSPCs were electroporated using the Neon Electroporator (ThermoFisher Scientific) with the 10 μl Neon Tips at 1700V, 20 ms and 1 pulse. The cells were carefully transferred directly to pre-warmed fresh media and culture for 48 hours.

4 × 10^6^ *ex vivo* expanded human HSPCs were collected via centrifugation and resuspended in 50 μl of SFEM II media. The cells were then transferred into the tip of the syringe connector. Then the mixture is forced through the syringe and membrane via 5 PSI of pressure. The flow-through was allowed to rest for approximately 2–5 minutes while the membrane was washed with 1 ml of complete media. The cells were then supplemented with fresh media and cultured for 48 hours.

After 48 hour recovery of both conditions, 5 × 10^4^ viable HSPCs were seeded from each condition and expanded for 7 days, and then counted by Vi-CELL and analyzed by CD34/CD90 staining via FACS.

#### NGS Analysis

PCR amplicons were purified using 1.8x Agencourt AmpureXP beads (Beckman Coulter) following the manufactures recommendations. Amplicons were quantified using the Quant-iT PicoGreen dsDNA assay (Life Technologies) following the manufacture’s recommendations. Illumina sequencing libraries were generated using the Nextera DNA Library Prep Kit (Illumina) following the manufacture’s recommendations with the following changes. Tagmentation was performed in a final volume of 5 μl using 5 ng of purified PCR product, 0.15 μl of Nextera tagment enzyme and tagmentation buffer previously described by Wang *et al*.^50^. Tagmented amplicons were then PCR amplified in a final volume of 50 μl using a final concentration of 0.2 mM dNTP (Life Technologies), 0.2 μM Illumina index PCR primers (Integrated DNA Technologies), 1x Phusion DNA polymerase buffer (New England Biolabs) and 1U of Phusion DNA polymerase (New England Biolabs). PCR cycling conditions used were as follows: 72 °C for 3 minutes, 98 °C for 2 minutes and 15 cycles of 98 °C for 10 seconds, 63 °C for 30 secconds, and 72 °C for 3 minutes. Sequencing libraries were then purified using 1.0x Agencourt AmpureXP beads (Beckman Coulter) following the manufacture’s recommendations. Sequencing libraries were quantified using the Quant-iT PicoGreen dsDNA assay (Life Technologies) following the manufacture’s recommendations and pooled equimolar for sequencing. Sequencing libraries were sequenced with 150 base paired-end reads on a MiSeq sequencer following the manufacture’s recommendations (Illumina). A minimum of a 1000-fold sequencing coverage was generated per amplicon.

Reads generated by the standard MiSeq reporter software (version 2.6.2, Illumina) were aligned to the human genome reference sequence (build GRCh38) with the BWA-MEM aligner (version 0.7.4-r385)^51^ using ‘hard-clipping’ to trim 3′ ends of reads of Illumina sequences and low quality bases. Resulting reads were aligned a second time but this time without ‘hard-clipping’. Reads were then subjected to variant calling using the VarDict variant caller (version 1.0 ‘Cas9 aware’ modified by developer ZhongWu Lai)^52^ with the allele frequency detection limit set at >= 0.0001. Variants identified were filtered for known variants found in dbSNP^53^. Variants in the edited samples were further filtered to exclude variants identified in the unedited controls, variants with a VarDict strand bias of 2:1, variants located >5 bp away from the Cas9 cut site (upstream or downstream), and single nucleotide variants.

#### qPCR Detection of Large HbF Deletions

To detect large 4.9 kb deletions, a primer and probe set was designed to detect the amplification of an HBG1 promoter specific sequence^15^. HBG Fwd: ACGGATAAGTAGATATTGAGGTAAGC, HBG Rev: GTCTCTTTCAGTTAGCAGTGG, and TaqMan probe (FAM): ACTGCGCTGAAACTGTGGCTTTATAG. TaqMan qPCR was performed directly on the lysate from CD34+ cells using the Universal TaqMan Mix (Thermo Fisher Scientific). Average ΔCT values were calculated by subtracting average CT value of HbG minus average CT of RPPH1 (Thermo Fisher Scientific), for copy-number reference, for each gDNA samples and the ΔΔ Ct values were calculated by subtracting ΔCT value of edited sample minus average untreated ΔCT. Fold was calculated by taking (2^−ΔΔCT^).

#### Antibody Staining to Detect B2M Knockout

Functional editing was determined by assessing B2M knockout via FACS analysis. Cells were collected via centrifugation and stained with FITC conjugated B2M antibody (BioLegend, Clone 2M2) and with Human TruStain FcX for 30 minutes (BioLegend). The cells were washed twice and then analyzed with BD Fortessa Flow Cytometer (Becton Dickinson).

#### Cell Recovery

Cell recovery was determined by running 500 μl of the cells 72 hours post transfection in the Beckman Coulter Vi-Cell to count viable cells. The cell recovery was determined by comparing to an untreated cell sample size with the same number of cells seeded in the same volume via this equation:

$${Cell}\,{Recovery}\,\, \% =\frac{(\#viable\,cells\,of\,sample)}{average(\#viable\,cells\,from\,untreated\,sample)}\cdot $$

#### Erythroid Differentiation and HbF Detection

Erythroid differentiation was performed following StemCell Technologies’ two phase method. Briefly, cells were cultured at 10k cells/ml in SFEM II supplemented with Erythroid Expansion Supplement (02692) and 100 U/ml Penicillin-Streptomycin (LifeTech 15140-122) for 10 days. After 10 days, the cells were washed once with PBS, and then cultured in SFEM II supplemented with 3% human AB serum, 3 U/ml of recombinant human EPO, and 100 U/ml Penicillin-Streptomycin (LifeTech 15140-122). Cells were cultured for an additional 11 days. After 4 and 11 days, cells were collected, fixed, permeabalized, and stained with APC conjugated anti-CD71 (BioLegend, clone CY1G4), FITC conjugated anti-GlyA (CD235a) (BioLegend, clone HI264), and PE conjugated anti-HbF (Invitrogen, clone HBF-1).

#### CFU Assay

Colony forming assay was conducted with Methocult Optimum (Stem Cell Technologies) according to manufacturer’s instructions. Briefly, cells were counted, diluted, and plated at 300 cells per 6-well well. After 14 days, colonies were counted and scored automatically with the StemVision system (StemCell Technologies).

#### *In-Vivo* Engraftment

NSG mice were purchased from The Jackson Laboratory (Stock #005557) and housed in a 12 hr light/dark cycle facility and had access to food and *ad libitum*. All experiments involving animals were approved by the NIBR IACUC (protocol #15DMP047) and were conducted in full compliance to NIBR IACUC guidelines.

Bone marrow derived CD34^+^ HSPCs cells from a single donor were cultured for 2 days under the aforementioned expansion conditions. At day 3, 50 μl of 5 million HSPCs were transduced with 25 μM of RNP containing the g1-RNA by TRIAMF using 7 μm thick membrane with 8 μm pore diameter at 5 PSI. Three groups were prepared: untreated, TRAMF treated without RNP (TRIAMF Mock), and TRIAMF treated with RNP (TRIAMF + RNP). Cells were cultured in the expansion medium for one day after TRIAMF. At day 4, the cells were washed once with PBS and counted. Six to eight week old female NSG mice were irradiated 4 hr prior to transplantation with 200 Rad using a Cesium irradiator. 7 × 10^5^ live cells from each group were transplanted into each recipient mouse via tail vein injection. Following transplantation, the mice were placed on an antibiotic regimen for 4–6 weeks. At weeks 8, 12, 16 and 20 post injection, peripheral blood samples were collected via retro-orbital bleeding, RBCs were lysed with ACK buffer (Lonza, 10-548E), and then stained with anti-human and mouse CD45 antibodies to quantify human cell engraftment.

At week 20 post transplantation, mice were euthanized and bones (2x femur, 2x tibia, 2x iliac crest, and spine) were collected and crushed using mortar and pestle. RBCs were then lysed with ACK buffer. A fraction of the cells were stained BV510-human CD45 (Biolegend, clone: HI30, 304036), BV421-mouse CD45 (Biolegend, clone: 30-F11, 103134), Alexa700-CD3 (Biolegend, clone: OKT3, 317340), AF488-CD19 (Biolegend, clone: HIB19, 302219), APC-CD33 (Biolegend, clone: P67.6, 366606), for lineage markers. Another fraction of cells were stained for human CD45, mouse CD45 and human CD34 (Biolegend, clone: 561, 343620) for hematopoietic stem cell markers. Cells were also FACS sorted to isolate human CD34 cells for NGS and HbF analysis.

## Electronic supplementary material

Supplementary data
