# Supplementary material for: TRIAMF: A New Method for Delivery of Cas9 Ribonucleoprotein Complex to Human Hematopoietic Stem Cells
Source: Sci Rep. 2018 Nov 2;8:16304. doi: 10.1038/s41598-018-34601-6 (PMC6214993; doi:10.1038/s41598-018-34601-6)
Supplement: Supplementary file 1 — Supplementary data [file 41598_2018_34601_MOESM1_ESM.pdf]

# TRIAMF: A New Method for Delivery of Cas9 Ribonucleoprotein Complex to Human Hematopoietic Stem Cells

Jonathan Yen[1], Michael Fiorino[2], Yi Liu[1], Steve Paula[1], Scott Clarkson[1], Lisa Quinn[3], William R. Tschantz[3], Heath Klock[4], Ning Guo[1], Carsten Russ[1], Vionnie W.C. Yu[1], Craig Mickanin[1], Susan C. Stevenson[1], Cameron Lee[5], and Yi Yang[1,6]

Figure S1. Representative distribution of diameters of untreated and 2-day post TRIAMF treated cells measured by Vi-CELL XR cell counter. Representative distribution of the forward scatter of the CD34+ and CD34+/CD90+ cells that were untreated, treated with TRIAMF in the absence of RNP (TRIAMF Mock) or presence of RNP (TRIMAF+RNP).

Figure S2. B2M knockout efficiency in total, $CD34^+$ or $CD34^+/CD90^+$ populations of untreated, mock treated (TRIAMF Mock), and TRIAMF+RNP treated HSPCs.

Figure S3. B2M knockout efficiency determined by NGS as a function of (a) membrane pore diameter; (b) applied pressure; (c) RNP concentration and (d) cell density (n=6, 3 donors with duplicate of each donor). (e) B2M knockout efficiency remained the same with further increased cell density from $10^8$ to $2x10^8$ cells/ml in 50 μl volume processed by TRIAMF using 25 μM of RNP, 7 μm thick membrane with 8 μm pore diameter under 5 PSI nitrogen pressure (n=2). The samples are identical to the ones in Fig.1. *** $p < 0.001$, ** $p < 0.01$, one-way analysis of variance (ANOVA) and Tukey's multiple comparison test.

Figure S4. HSPC recovery rates after TRIAMF or electroporation in the absence of RNP. $5x10^4$ viable cells seeded 48 hours post treatment and expanded for 7 days. Percentage of $CD34^+$ and $CD34^+/CD90^+$ are shown in (a) and the total cell number and the number of either $CD34^+$ or $CD34^+/CD90^+$ cells are shown in (b). n=4 single donor, biological duplicate with technical duplicate each, ** $p < 0.01$, one-way analysis of variance (ANOVA) and Tukey's multiple comparison test. Bars represent standard deviation.

Figure S5. Representative gating scheme of $CD235a^+/CD71^{-/low}$ cells after 3 weeks erythroid differentiation/maturation in experimental groups used in Fig. 2 and 3.

Figure S6. B2M knockout efficiency as determined by NGS (a) and cell recovery (b) of untreated, mock electroporated (Neon Mock) and RNP electroporated (Neon+RNP) HSPCs. 0.14 million of ex vivo expanded HSPCs were mixed with 1 μM RNP, brought to 10 μl final volume with Buffer T and electroporated with the Neon electroporator using the same conditions as in Fig. S4.

S1

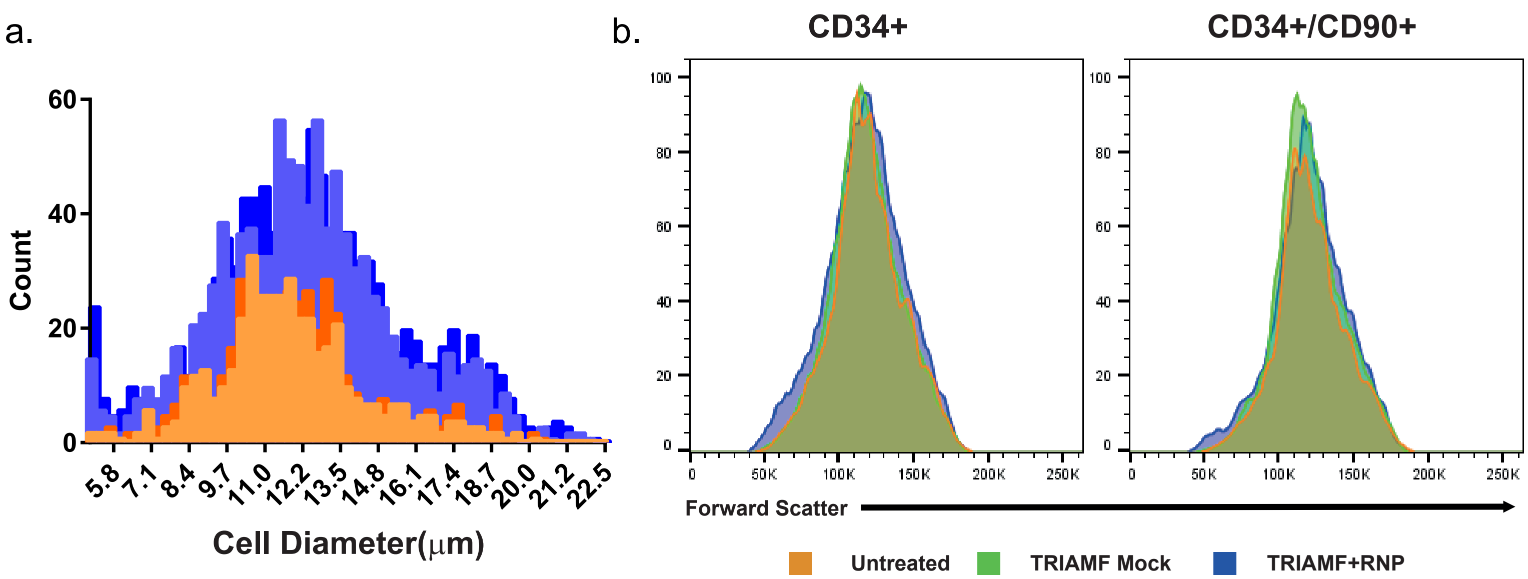
a.
Count
60
40
20
0
5.8
7.1
8.4
9.7
11.0
12.2
13.5
14.8
16.1
17.4
18.7
20.0
21.2
22.5
Cell Diameter(μm)
b.
CD34+
CD34+/CD90+
100
80
60
40
20
0
50K
100K
150K
200K
250K
Forward Scatter
Untreated
TRIAMF Mock
TRIAMF+RNP

S2

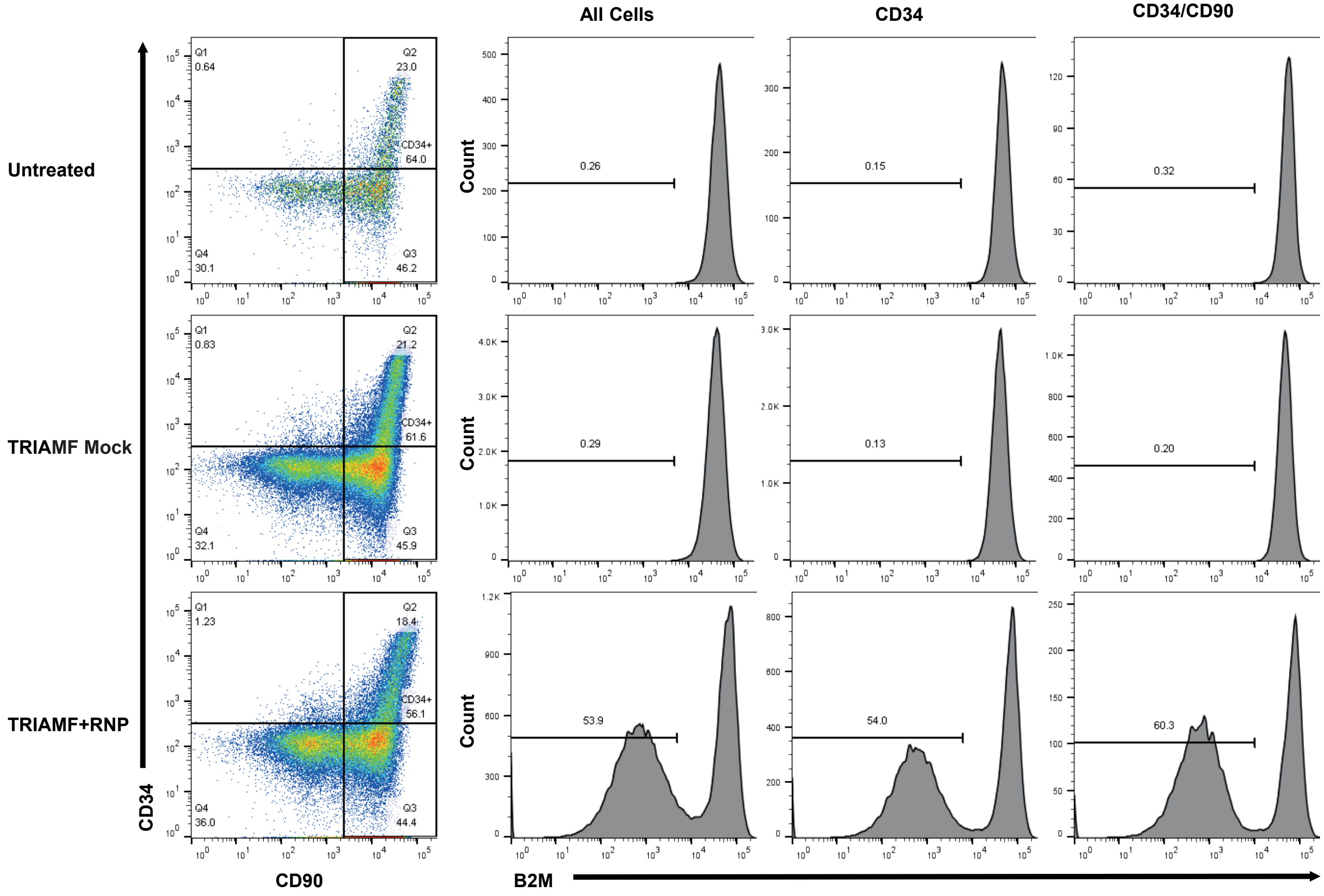
All Cells
CD34
CD34/CD90
Untreated
TRIAMF Mock
TRIAMF+RNP
Q1
0.64
Q2
23.0
CD34+
64.0
Q4
30.1
Q3
46.2
Q1
0.83
Q2
21.2
CD34+
61.6
Q4
32.1
Q3
45.9
Q1
1.23
Q2
18.4
CD34+
56.1
Q4
36.0
Q3
44.4
0.26
0.15
0.32
0.29
0.13
0.20
53.9
54.0
60.3
Count
CD34
CD90
B2M

S3

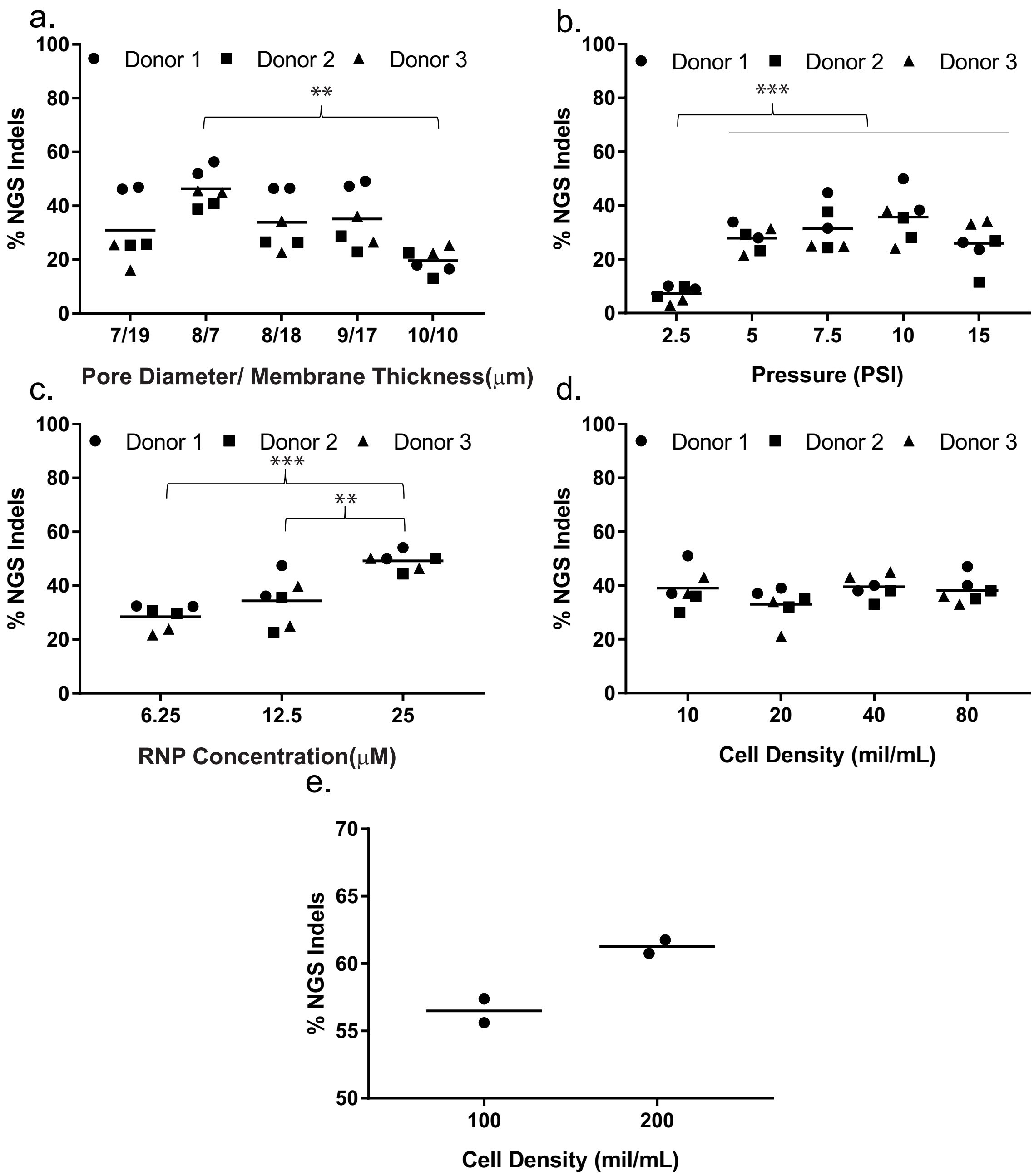
a.
% NGS Indels
Donor 1
Donor 2
Donor 3
**
100
80
60
40
20
0
7/19
8/7
8/18
9/17
10/10
Pore Diameter/ Membrane Thickness(μm)
b.
% NGS Indels
Donor 1
Donor 2
Donor 3
***
2.5
5
7.5
10
15
Pressure (PSI)
c.
% NGS Indels
Donor 1
Donor 2
Donor 3
***
**
6.25
12.5
25
RNP Concentration(μM)
d.
% NGS Indels
Donor 1
Donor 2
Donor 3
10
20
40
80
Cell Density (mil/mL)
e.
% NGS Indels
70
65
60
55
50
100
200
Cell Density (mil/mL)

S4

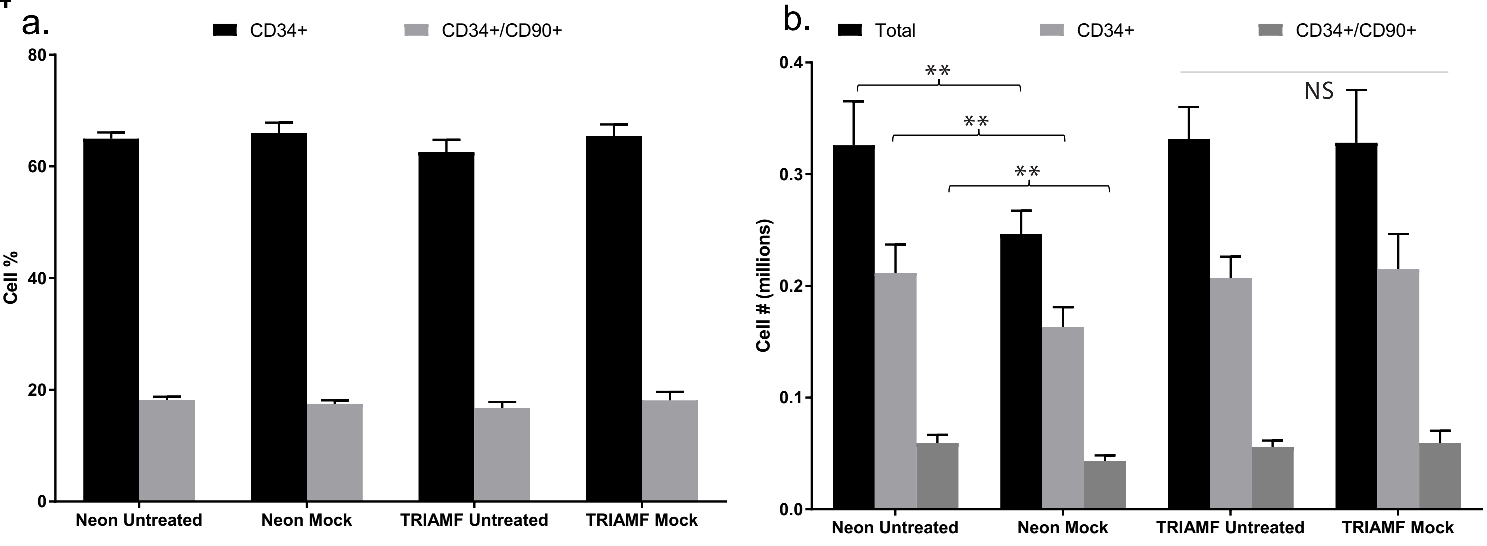
a.
CD34+
CD34+/CD90+
Cell %
80
60
40
20
0
Neon Untreated
Neon Mock
TRIAMF Untreated
TRIAMF Mock
b.
Total
CD34+
CD34+/CD90+
Cell # (millions)
0.4
0.3
0.2
0.1
0.0
**
**
**
NS
Neon Untreated
Neon Mock
TRIAMF Untreated
TRIAMF Mock

S5

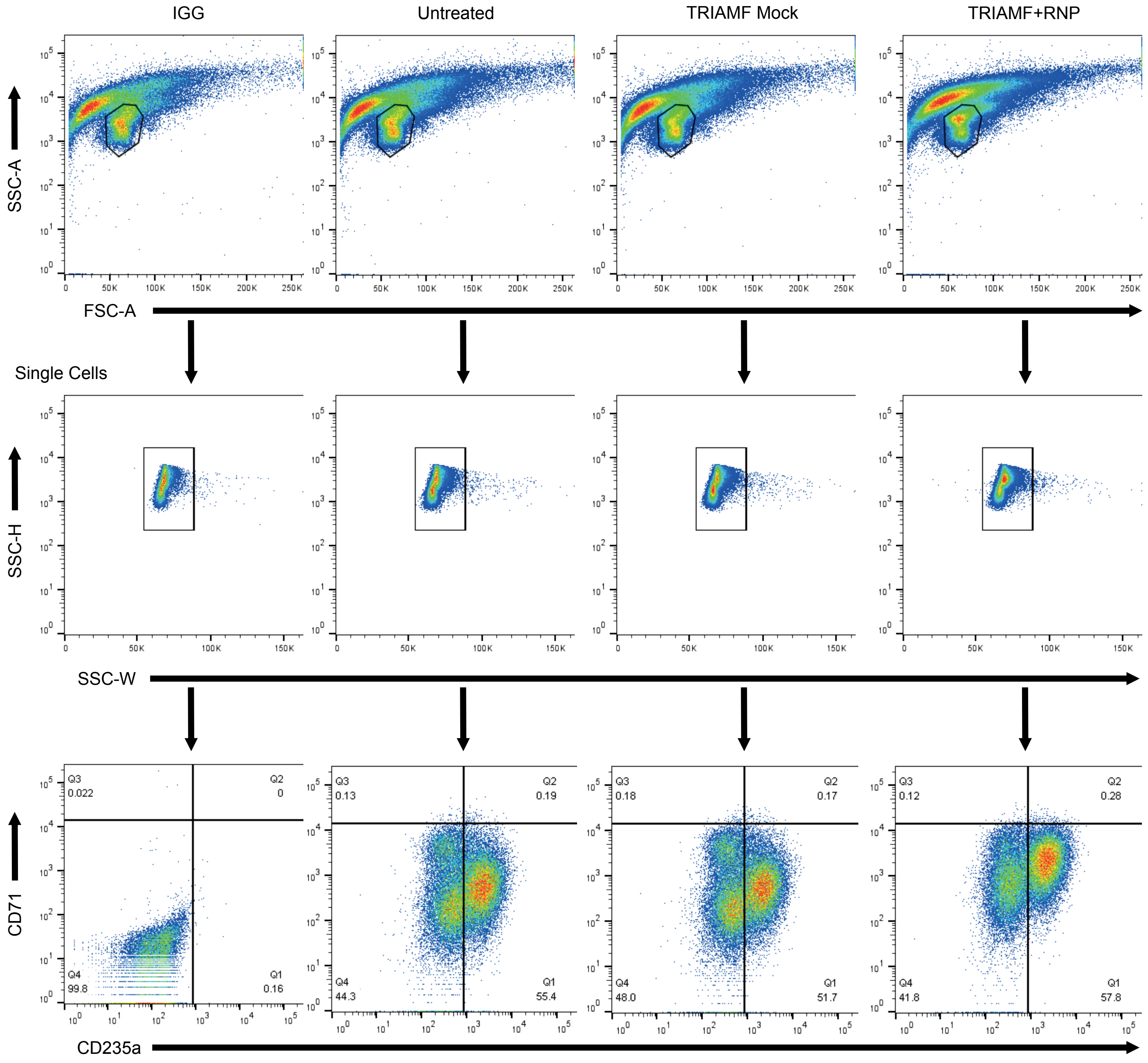
IGG
Untreated
TRIAMF Mock
TRIAMF+RNP
SSC-A
FSC-A
Single Cells
SSC-H
SSC-W
CD71
CD235a
Q3 0.022
Q2 0
Q4 99.8
Q1 0.16
Q3 0.13
Q2 0.19
Q4 44.3
Q1 55.4
Q3 0.18
Q2 0.17
Q4 48.0
Q1 51.7
Q3 0.12
Q2 0.28
Q4 41.8
Q1 57.8

S6

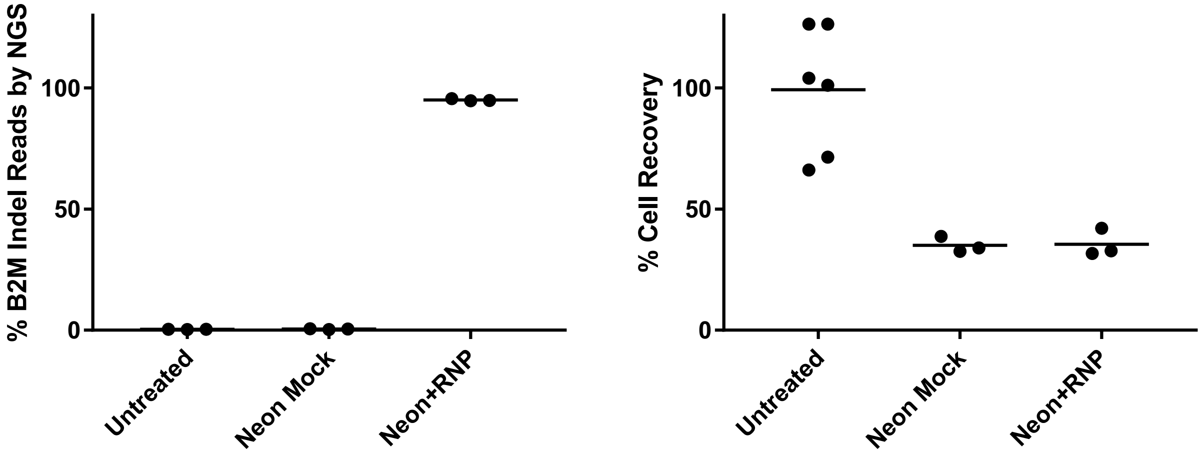
% B2M Indel Reads by NGS
% Cell Recovery
100
50
0
Untreated
Neon Mock
Neon+RNP
